# Spike-Timing-Dependent Plasticity: A Comprehensive Overview

**DOI:** 10.3389/fnsyn.2012.00002

**Published:** 2012-07-12

**Authors:** H. Markram, W. Gerstner, P. J. Sjöström

**Affiliations:** ^1^Brain Mind Institute Life Science, Ecole Polytechnique Federale de LausanneLausanne, Switzerland; ^2^Ecole Polytechnique Federale de LausanneLausanne, Switzerland; ^3^Department of Neurology and Neurosurgery, Centre for Research in Neuroscience, The Research Institute of the McGill University Health CentreMontreal, QC, Canada

## Why Timing Matters

A neuron embedded in a neuronal network is bombarded with thousands of inputs every minute. But which ones are important? Which information should the neuron listen to and pass along to downstream neurons?

During brain development and during learning, this is a formidable problem that the vast majority of neurons in the brain have to solve – how to correctly choose and fine-tune the inputs from one's neighbors without any other information than that which is received from these neighbors themselves. Some of these neighbors provide good information while others do not. Who to trust? How can you determine who to pay attention to?

Many decades ago, the Canadian neuropsychologist Hebb (1949), the Polish neurophysiologist Konorski (1948), as well as the Spanish anatomist Ramón y Cajal (1894) all had similar ideas that could potentially help explain how neurons wire up. The basic idea was essentially that – in the words of the present-day neuroscientist Shatz (1992) – “*cells that fire together, wire together*.” In other words, if things keep happening more or less simultaneously, you may assume that there is a common cause for the firing. More importantly, if one of the cell is active systematically just slightly before another, the firing of the first one might have a causal link to the firing of the second one and this causal link could be remembered by increasing the wiring of connections, a notion we call synaptic plasticity. In short, timing matters because it may indicate causality.

Even though Hebb did propose an ordering of firing in his “phase sequences” (Hebb, 1949), the view that coincident activity in connected neurons is what matters in plasticity practically dominated modern neuroscience research well into the mid 1990s. Although some few studies had indeed been carried out prior to this (e.g., Levy and Steward, 1983), neuroscientists typically did not consider the precise timing of inputs in their synaptic plasticity experiments. But this changed rapidly when a flurry of studies were published in the mid 1990s. Theoreticians realized just how important temporal order was for conveying and storing information in neuronal circuits, and to hook them up correctly. And experimenters realized that they had almost completely ignored this one factor – time – in their experiments, while they at the same time saw how the synaptic connections of the brain had mechanisms in place that clearly should make them acutely sensitive to timing. Thus the field of Spike-Timing-Dependent Plasticity – or STDP – was born, via the first key studies of Henry Markram (Markram and Sakmann, 1995; Markram et al., 1997) and Gerstner et al. (1996).

With STDP, a neuron embedded in a neuronal network can determine which neighboring neurons are worth listening to by potentiating those inputs that predict its own spiking activity. However, the neuron in question pays less attention to those neighboring neurons that fail to do this. In other words, the neuron pays less attention to neighbors speaking gibberish. The net result is that our sample neuron can integrate inputs with predictive power and transform this is into a meaningful predictive output, even though the meaning itself is not strictly known by the neuron. In STDP we thus have a very simple and elegant algorithm for appropriately hooking up neurons in the brain. Little wonder that there has been so much excitement surrounding the discovery of STDP.

## The Stdp Research Topic: A Brief Introduction

This Frontiers Research Topic eBook has been divided into eight sections, of which six contain reviews and two comprise original research articles. The first section is called *The Conceptual Development of STDP*, and deals with the history leading up to the discovery of STDP. Markram et al. (2011) outlines the history of timing in plasticity, beginning with Aristotle some 2000 years ago. Sjöström and Gerstner (2010) next define and briefly outline the STDP concept. Because the history of anything is necessarily subjective, this section also includes personal accounts, published as Opinion pieces. Contributors include the Nobel prize winner Cooper (2010), who famously helped outline the Bienenstock–Cooper–Munro theory of metaplasticity (Bienenstock et al., 1982); the electrophysiologist Levy (2010), who arguably carried out some of the very first timing-dependence experiments in plasticity (Levy and Steward, 1983); and the theoretician Gerstner (2010), who in a theoretical study (Gerstner et al., 1996) independently predicted and anticipated Henry Markram's report of STDP (Markram and Sakmann, 1995; Markram et al., 1997).

In the second section, *The Biological Relevance of STDP* is discussed. Lisman and Spruston (2010) are first out and argue that STDP is limited plasticity paradigm that cannot unify the field of plasticity, because its biological relevance is overrated. This is an important criticism – which the authors have made before (Lisman and Spruston, 2005) – that researchers in the STDP field should take to heart and try to address. Schulz (2010) develops this point, making the case that plasticity in the intact brain is likely to be much more complicated than in simple *in vitro* experiments. In a more specific argument, Shouval et al. (2010) suggest that STDP is in reality a result of something more fundamental, which they propose is intracellular calcium signals. In addition, they argue in favor of mechanism-driven modeling, rather than theory driven by phenomenology. Fregnac et al. (2010) make the case that there is limited evidence supporting an actual functional role of STDP in the intact brain, an important point that should be compared to those of Schulz (2010) and of Lisman and Spruston (2010). By comparing two different induction protocols, they conclude that classical STDP might be limited to the critical period *in vivo*. Finally, Buchanan and Mellor (Buchanan et al., 2010) focus on STDP in the hippocampus, showing that for this brain region the experimental literature seems to be particularly fraught with disagreement. But there is common ground, which is defined by post-synaptic calcium transients, thus echoing the point made by Shouval et al. (2010).

Section three deals with *Mechanisms: Inducing, Expressing, and Controlling STDP*. Seguing from the previous section, Graupner and Brunel (2010) begin by providing an overview of biophysical models of synaptic plasticity, including those based on calcium and those based on signaling cascades, with a special emphasis on bistable synapses. Following on this, Froemke et al. (2010b) explore how STDP depends on where the synapse is located in the dendritic arbor, and what the functional consequences of this location dependence might be (for a related article here-in, see Clopath and Gerstner, 2010). Turning next to the pre-synaptic side and how it governs STDP, Rodriguez-Moreno et al. (2010) overview recent findings on the role of pre-synaptically located NMDA receptors in timing-dependent long-term depression (cf. Duguid and Sjöström, 2006). But pre or post-synaptic mechanisms cannot suffice – as Pawlak et al. (2010) show in the ensuing paper, STDP must be somehow controlled by a third factor, which is likely a neuromodulatory gate. Finally, Froemke et al. (2010a) discuss the consequences of temporally non-linear spike-pair interactions in STDP. They show that factors such as spike triplets and rate also determine plasticity, although differently in neocortex compared to hippocampus.

This brings us to the next set of questions: Does STDP always look the same? In fact, has STDP been found at all synapse types? In section four, these and other questions are addressed as we learn about *The Diverse Phenomenology of STDP*. Shulz and Jacob (2010) compare STDP in different species and brain regions, in particular *in vivo*, and find that variability depends not only on synapse type, but also on network state and neuromodulation, thus arguing for the need for more research. Müller-Dahlhaus et al. (2010) report on STDP-like changes in the human brain as evidenced by transcranial magnetic stimulation. Richards et al. (2010) subsequently report on *in vivo* STDP in the optic tectum of the tadpole, *Xenopuslaevis*, where some of the first evidence for the existence of STDP was found. So far, we have focused on the plasticity of excitation, but it is important not to neglect the plasticity of inhibitory circuits, as is pointed out in the review by Lamsa et al. (2010). After this, Roberts and Leen (2010) discuss the role of anti-Hebbian STDP in computational features such as predictive sensory cancelation and novelty detection in the electrosensory system of the weakly electric fish. Next, Fino and Venance (2010) overview the state of the striatal STDP field, where some conflicting results have been reported. The authors argue that these discrepancies are due to diversity in synaptic learning rules across different cell types, but probably also to experimental conditions. Finally, Larsen et al. (2010) review STDP in the sensory neocortex, arguing that the properties of STDP change over development, to achieve optimal tuning of neurons as conditions change with maturation. To conclude, STDP exists in humans, tadpole, and electric fish alike, but it may vary with the specific cell and synapse type as well as with developmental stage.

Timing is not everything, however, and in section five, titled *STDP and Beyond*, we learn about other forms of plasticity and how they interact with timing-dependent learning rules. Watt and Desai (2010) discuss how homeostatic plasticity is necessary for a neuron to keep its cool as the world changes, for example as inputs connect up during development. Such homeostatic plasticity may act on synapses or on the excitability of the cell itself. Debanne and Poo (2010) go beyond the synapse to the plasticity of intrinsic excitability of the pre or the post-synaptic cell, overviewing how this is linked to the induction of STDP, as well as what its spatial extent is.

In section six, entitled *STDP: Consequences in Health and Disease*, the impact of STDP on circuits is discussed. Butts and Kanold (2010) make the interesting argument that – because early activity patterns typically do not possess the fast correlation structures that STDP is sensitive to, but mature activity patterns do – STDP may first be masked, only to emerge later in development and be present in the mature brain (for a different view, see Fregnac et al., 2010). Next up is Gilson et al. (2010), who explore STDP in recurrent neuronal networks. Although several theoretical studies have explored the role of STDP in individual cells, Gilson et al. (2010) discuss the consequences of STDP in the circuit, with a focus on weight dynamics and the evolution of different network structures. Finally, Meredith and Mansvelder (2010) overview STDP in neurodevelopmental learning disorders, using the Fragile X syndrome as a starting point. They propose that studying STDP in a disease context may provide an opportunity to link cognition and learning rules.

The two last sections consist of original research articles grouped into theoretical and experimental studies. Due to lack of space, we regrettably cannot introduce these contributions individually here.

## Concluding Remarks

There is no doubt that STDP is a novel plasticity paradigm of great interest that holds particular promise for biological and computational relevance. STDP has in fact dramatically reshaped the field of synaptic plasticity over the past decade or so. But this is not to say that STDP has been a panacea for all problems neuroscientific. Clearly cells that fire together wire together, there is no doubt about that, so the coming of STDP has not rendered the classical literature obsolete by any means. Also, the advent of STDP has raised many questions. Can we really be sure that STDP actually happens in the intact brain? Most studies have been carried out in a dish, after all, so we should not assume that these artificial activity patterns imposed on the tissue in the dish are necessarily relevant. If STDP does exist, does it exist in all animal species? After all, if STDP is inherently important for brain functioning, it should have been relatively preserved by evolution. And should there not be a way of turning STDP on and off? It does not seem to be the case that we constantly learn and rewire our brains to every stimulus we encounter; our brains are quite selective filters when it comes to information storage. If the STDP paradigm shift is to be more than a revolution in a dish, a much-improved understanding of this phenomenon is desperately and urgently needed. This Frontiers Research Topic eBook on STDP has aimed to help achieve precisely this, by comprehensively overviewing what is known, outlining what is not known, highlighting controversy, and pointing out where we need to direct our research efforts next. This research is important, because it is about how your brain works.
